# Translation and Validation of the Malay Doctor–Patient Communication Questionnaire: A Cross-Sectional Study Among Patients Receiving Hemodialysis in Kelantan, Malaysia

**DOI:** 10.3390/healthcare13162037

**Published:** 2025-08-18

**Authors:** Ab Farid Fajilah Ab Aziz, Mohd Ismail Ibrahim, Najib Majdi Yaacob, Afiq Izzudin A Rahim

**Affiliations:** 1 Department of Community Medicine, School of Medical Sciences, Universiti Sains Malaysia, Kubang Kerian, Kota Bharu 16150, Kelantan, Malaysia; abfarid21@student.usm.my (A.F.F.A.A.); drafiqrahim@usm.my (A.I.A.R.); 2 Units of Biostatistics and Research Methodology, School of Medical Sciences, Health Campus, Universiti Sains Malaysia, Kubang Kerian, Kota Bharu 16150, Kelantan, Malaysia; najibmy@usm.my

**Keywords:** doctor–patient communication, MyD-PCQ, hemodialysis, cross-sectional study, translation and validation, Malaysia

## Abstract

**Background**: Effective doctor–patient communication is essential for high-quality care, especially for patients with chronic conditions requiring hemodialysis. However, there is a lack of validated tools in the Malay language to measure this communication. This study aimed to translate and validate the Doctor–Patient Communication Questionnaire (DPCQ) into Malay (MyD-PCQ) for use among patients receiving hemodialysis in Kelantan, Malaysia. **Methods**: A cross-sectional study was conducted with 300 patients receiving hemodialysis at Hospital Universiti Sains Malaysia. The original English DPCQ was translated and culturally adapted into Malay following international guidelines, including forward and backward translation, expert review, and cognitive debriefing. Data were collected using the Malay version of the questionnaire. Confirmatory factor analysis (CFA) assessed the construct validity, while Raykov’s rho measured internal consistency. **Results**: The Malay version of the DPCQ demonstrated excellent model fit in CFA (χ^2^/df = 1.25, *p* = 0.053; SRMR = 0.037; RMSEA = 0.029; CFI = 0.982; and TLI = 0.979). Factor loadings ranged from 0.493 to 0.640. The internal consistency was high, with Raykov’s rho of 0.887. The average total score among participants was 37.31 out of 60, indicating moderate perceived communication quality. **Conclusions**: The Malay Doctor–Patient Communication Questionnaire (MyD-PCQ) is a valid and reliable tool for assessing communication between doctors and patients receiving hemodialysis in Malaysia. Its use can help identify communication gaps, support training initiatives, and improve patient-centered care in clinical practice. Future research should evaluate its use in other settings and patient populations.

## 1. Introduction

Doctor–patient communication is widely recognized as a fundamental component of effective healthcare delivery, forming the basis for trust, shared decision-making, and high-quality patient care [1,2,3,4]. When communication is clear and respectful, patients are more likely to understand their conditions, follow treatment recommendations, and engage actively in their care, leading to better clinical outcomes and greater satisfaction. This is especially important for patients with chronic diseases who require sustained interactions with their healthcare teams over long periods [1,2,3,4].

Patients receiving hemodialysis represent one of the most vulnerable groups in this context. These patients experience life-altering chronic kidney disease that requires complex treatment decisions, strict adherence to demanding regimens, and frequent hospital visits. The psychological burden, physical side effects, and social disruptions associated with hemodialysis can significantly affect patients’ quality of life, making effective communication with healthcare providers essential [5,6,7,8]. Good doctor–patient communication can help alleviate patients’ anxieties, improve trust in medical advice, and promote collaborative decision-making that respects patient preferences and values. Conversely, communication failures can result in misunderstandings about treatment plans, decreased adherence, dissatisfaction, avoidable complications, and even preventable harm [9,10,11].

The construct of doctor–patient communication encompasses both informational and relational components, including active listening, empathy, mutual respect, and shared decision-making. Theoretical frameworks such as the Relational Communication Model and Shared Decision-Making Theory highlight that effective communication not only conveys information but also builds a therapeutic alliance and trust between doctors and patients [4,7,8]. In chronic disease contexts such as hemodialysis, this construct is particularly salient due to the long-term, repetitive interactions between patients and healthcare providers, where consistent, respectful, and clear communication can enhance adherence, reduce anxiety, and improve patient satisfaction and outcomes [5,6].

Despite these critical needs, communication challenges remain pervasive in hemodialysis care. Studies report that patients often feel their concerns are not heard, experience difficulty understanding medical explanations, or perceive that their input is not valued in decision-making [12,13]. As the number of patients requiring hemodialysis continues to increase in Malaysia, there is an urgent need to ensure that healthcare providers are equipped with the skills and tools necessary to deliver patient-centered communication that meets these complex needs [14,15,16,17,18].

However, the assessment and improvement of doctor–patient communication in Malaysia face significant challenges. Research on communication quality among patients receiving hemodialysis remains limited, with most existing evidence coming from Western settings with different cultural, linguistic, and healthcare system contexts [19,20,21,22,23]. In Malaysia, local research has primarily focused on clinical outcomes, with insufficient attention to the relational and communicative aspects of care [24,25,26]. Without contextually appropriate evidence, it is difficult for policymakers and healthcare providers to develop targeted interventions to strengthen communication in hemodialysis care.

Critically, there is currently no validated instrument in the Malay language designed specifically to measure doctor–patient communication among patients receiving hemodialysis. Tools developed elsewhere may fail to capture cultural nuances, linguistic expressions, and patient expectations unique to Malaysia, limiting their validity and practical utility. A culturally adapted and rigorously validated questionnaire is essential to fill this gap. Such a tool would allow healthcare providers and researchers to systematically assess communication quality, identify strengths and weaknesses in practice, and implement targeted training and service improvements to enhance patient-centered care [27,28,29,30].

While several instruments exist to assess doctor–patient communication, the Doctor–Patient Communication Questionnaire (DPCQ) was selected for its concise structure, broad coverage of essential communication behaviors, and previous validation in acute care and chronic disease contexts [27,31]. Compared to alternatives such as the Trust in Physician Scale [30] or the Communication Assessment Tool [29], the DPCQ captures both interpersonal and informational dimensions within a single construct, making it particularly suited for evaluating the complex communication needs of patients receiving hemodialysis. Moreover, it offers strong psychometric evidence and has demonstrated applicability in multicultural healthcare environments [31].

To address these gaps, this study aimed to translate and validate the Doctor–Patient Communication Questionnaire (DPCQ), originally developed by Sustersic et al. [31], into Malay, resulting in the MyD-PCQ. By establishing its validity and reliability among patients receiving hemodialysis in Kelantan, Malaysia, this study provides a robust, culturally appropriate tool that can support improved communication practices, guide training efforts, and ultimately enhance the quality of care for patients with chronic kidney disease [32,33,34,35,36,37]. Based on the theoretical model and previous validations, we hypothesized that the Malay version of the DPCQ (MyD-PCQ) would demonstrate a unidimensional factor structure with good construct validity and high internal consistency reliability, supporting its use in the Malaysian hemodialysis setting.

## 2. Materials and Methods

### 2.1. Study Design and Setting

This study employed a cross-sectional design conducted over two months, beginning in June 2024, at the hemodialysis unit of Hospital Universiti Sains Malaysia (HUSM), Kelantan. The hemodialysis unit provides regular outpatient dialysis services for patients with end-stage renal disease (ESRD).

### 2.2. Participants and Sampling

Eligible participants were Malaysian adults aged 18 years or older, permanently registered for hemodialysis treatment at HUSM, who were able to understand and communicate in the Malay language and provided informed written consent.

Exclusion criteria included patients who were illiterate, unable to provide informed consent, or those with significant cognitive or psychiatric impairments that would hinder questionnaire comprehension and response.

The sample size of 300 participants was determined based on psychometric validation standards for confirmatory factor analysis (CFA). Following the general guideline of a minimum of 5 to 10 participants per item, and given that the DPCQ comprises 15 items, a sample of 150–300 participants was deemed appropriate [38]. The use of 300 participants ensures adequate power for factor extraction and enhances the stability and reliability of the model estimates.

### 2.3. Instrument Translation and Adaptation

The Doctor–Patient Communication Questionnaire (DPCQ) is a validated 15-item instrument, originally developed by Sustersic et al., measuring perceived doctor–patient communication on a 4-point Likert scale (1 = no to 4 = yes) [36]. The total score ranges from 15 to 60, with higher scores indicating better communication quality.

### 2.4. The Translation Process Followed the Guidelines Proposed by Beaton et al. [36]

Preparation: Identification of translators and timeline planning;

Forward Translation: Two independent bilingual translators (a public health specialist and a linguist) produced separate Malay versions;

Reconciliation: An expert committee reviewed both versions to create a single forward translation;

Back Translation: Two independent translators (a family medicine physician and an English teacher), blinded to the original, back-translated the reconciled Malay version into English;

Back-Translation Review and Harmonization: The committee compared back-translated and original versions for conceptual and semantic equivalence, finalizing the pre-final version;

Cognitive Debriefing: Ten participants (three patients receiving hemodialysis and seven clinical staff) completed and reviewed the pre-final Malay version, providing feedback on clarity, cultural relevance, and ease of understanding;

Finalization and Proofreading: The expert committee revised the translation based on feedback and prepared the final version for field testing.

### 2.5. Data Collection Procedures

Data collection was scheduled alongside patients’ hemodialysis sessions to minimize participant burden and ensure convenience. After obtaining informed consent, participants were given the final Malay-translated DPCQ to complete, taking approximately 20 min. A briefing session was provided to improve comprehension and reduce response errors.

### 2.6. Statistical Analysis

Data were entered into SPSS version 29 and imported into R version 4.4.1 for advanced analysis. Descriptive statistics summarized participants’ sociodemographic characteristics and DPCQ scores. To evaluate construct validity, confirmatory factor analysis (CFA) was conducted using robust maximum likelihood estimation [39,40]. Model fit was assessed using multiple recommended indices, including the chi-square to degrees of freedom ratio (with values ≤ 3 considered acceptable), *p*-values greater than 0.05 indicating good fit, comparative fit index (CFI) and Tucker–Lewis index (TLI) values of 0.95 or higher, root mean square error of approximation (RMSEA) of 0.08 or less with 90% confidence intervals, and standardized root mean square residual (SRMR) of 0.08 or less [41,42]. Factor loadings greater than 0.30 were considered acceptable [43]. Model selection was also guided by the Akaike information criterion (AIC) and Bayesian information criterion (BIC), with lower values indicating a better fit. Internal consistency reliability was evaluated using Raykov’s rho coefficient, with values ≥ 0.70 indicating acceptable reliability [43].

### 2.7. Ethical Approval

Ethical approval was obtained from the Universiti Sains Malaysia Human Research Ethics Committee (USM/JEPeM/KK/24020217).

## 3. Results

### 3.1. Participant Characteristics

A total of 300 patients receiving hemodialysis participated in this study. Table 1 summarizes their sociodemographic characteristics. The majority were female (64.7%) and over 58 years old (34.0%). Most participants identified as Malay (47.3%), were married (58.7%), and reported lower income levels (60.0%). Regarding education, 36.0% had completed O levels (equivalent to high school), while smaller proportions held A levels (pre-university qualifications) (26.7%), diplomas (16.0%), or degrees (e.g., bachelor’s or higher) (21.3%).

In terms of employment, 22.7% were housemakers, 22.0% were not working, and others were employed in government, private, or self-employed sectors. Participants generally rated the service provided positively: 87.3% agreed with and supported their treatment; 88.0% were satisfied with their doctor’s service; 89.3% felt their doctor was easily reachable; and 90.7% felt their doctor was ready to treat them.

### 3.2. Descriptive Statistics of Doctor–Patient Communication

Table 2 presents item-level descriptive statistics for the Malay Doctor–Patient Communication Questionnaire (MyD-PCQ). The average total score was 37.31 out of 60, indicating moderate perceived communication quality among participants.

Across items, most respondents selected a score of 3 (“possibly yes”) for doctor behaviors such as listening carefully, allowing patients to talk without interruption, encouraging expression, and explaining treatment plans. Mean scores for individual items ranged from approximately 2.45 to 2.52 (SD ~0.53–0.57), suggesting generally positive but not optimal communication ratings.

### 3.3. Confirmatory Factor Analysis Results

Confirmatory factor analysis (CFA) was conducted to assess the construct validity of the MyD-PCQ. Table 3 details the model fit indices. The CFA model demonstrated an excellent fit of χ^2^/df = 1.25, *p* = 0.053, indicating no significant deviation from the observed data. Other fit indices supported the model’s adequacy, with SRMR = 0.037, RMSEA = 0.029 (90% CI: 0.000–0.045), CFI = 0.982, and TLI = 0.979. Both the Akaike information criterion (AIC = 6187.240) and Bayesian information criterion (BIC = 6298.354) values supported the model’s stability.

**Figure 1:**
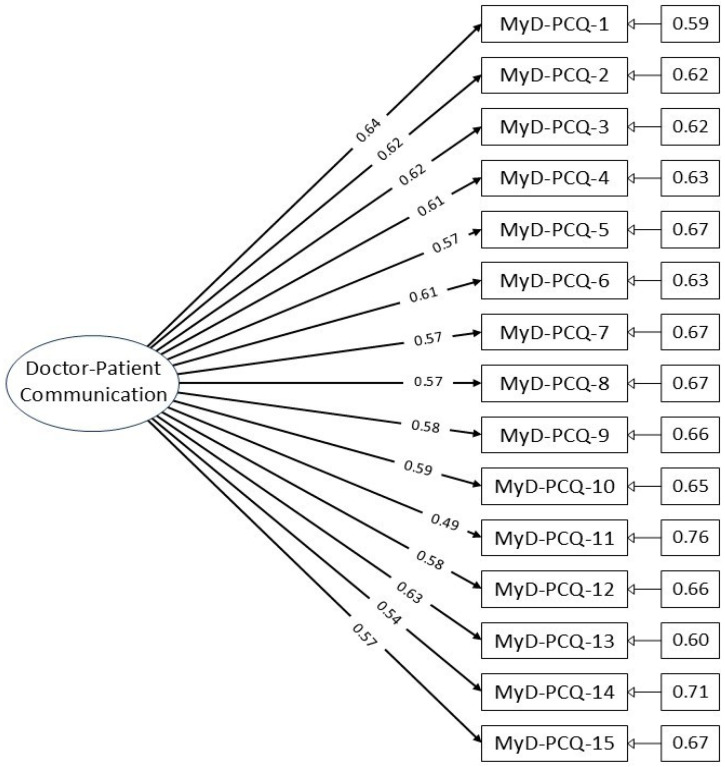
Path diagram of the MyD-PCQ confirmatory factor analysis model.

A CFA path diagram (Figure 1) illustrates the factor structure, showing all 15 items loading onto a single latent factor without cross-loadings or interaction terms, confirming the unidimensionality of the construct.

### 3.4. Factor Loadings and Reliability

Table 4 shows the factor loadings for individual items and the overall reliability of the scale. Item loadings ranged from 0.493 to 0.640, all exceeding the acceptable threshold of 0.30, indicating meaningful contributions to the latent construct.

The internal consistency reliability of the MyD-PCQ, measured by Raykov’s rho, was 0.887, demonstrating high reliability suitable for research and clinical use.

The inter-item correlation matrix (Supplementary Table S1) revealed moderate positive correlations between items, ranging from 0.217 to 0.456, indicating that items consistently reflected a common construct without excessive redundancy.

Assessment of skewness and kurtosis for each item (Supplementary Table S2) indicated acceptable normality, with skewness ranging between −0.155 and +0.213 and kurtosis ranging from −1.321 to −0.556. These values support the use of robust maximum likelihood estimation for confirmatory factor analysis.

## 4. Discussion

This study aimed to translate, culturally adapt, and validate the Doctor–Patient Communication Questionnaire (DPCQ) into Malay, resulting in the MyD-PCQ for use among patients receiving hemodialysis in Kelantan, Malaysia. The results provide strong evidence for the instrument’s validity and reliability, supporting its use to assess doctor–patient communication in this context.

The CFA demonstrated excellent model fit with all fit indices meeting or exceeding recommended thresholds, confirming the one-factor structure of the MyD-PCQ. These findings are consistent with the validation of the original DPCQ, which also demonstrated good construct validity in measuring the communication construct [44]. The robust fit indices in this study support the argument that the translated version maintains the theoretical framework of the original instrument while achieving cultural and linguistic appropriateness for Malaysian patients.

Item-level factor loadings in this study ranged from 0.493 to 0.640, all above the acceptable threshold of 0.30, indicating that each item meaningfully contributed to the overall construct of doctor–patient communication. This result aligns with previous psychometric research suggesting that well-constructed communication measures typically yield factor loadings above 0.40 [44]. Moreover, the internal consistency reliability was high (Raykov’s rho = 0.887), meeting the standard criteria for acceptable reliability (>0.70) and demonstrating that the items reliably measure a cohesive underlying construct.

The average MyD-PCQ total score of 37.31 out of 60 suggests that, while communication quality was generally positive, there remains room for improvement in hemodialysis care settings. Many participants gave “possibly yes” ratings rather than strong affirmative responses. This finding aligns with prior research highlighting that patients receiving hemodialysis often perceive barriers to fully open and participatory communication with their healthcare providers [37]. It underscores the importance of investing in training and system-level interventions to enhance communication skills among doctors treating patients with chronic kidney disease.

The translation process followed best-practice guidelines, ensuring conceptual, semantic, and experiential equivalence between the original and translated items [36]. Steps such as forward and backward translation, expert reconciliation, cognitive debriefing, and pilot testing helped achieve cultural adaptation, ensuring the tool is understandable and relevant to the Malaysian context. This rigorous process increases confidence in the MyD-PCQ’s suitability for clinical use and research in Malaysia.

The results of this validation are consistent with previous studies involving the DPCQ in other settings. For example, the original validation by Sustersic et al. demonstrated comparable internal consistency and a similar unidimensional structure, supporting the robustness of the instrument across diverse populations [31]. Other adaptations, such as those conducted in multilingual European healthcare contexts, also reported moderate to high factor loadings and satisfactory model fit indices [29,44]. Our findings, thus, reinforce the DPCQ’s cross-cultural adaptability and psychometric soundness when carefully translated and contextually adapted.

Beyond validation, the MyD-PCQ holds practical potential for use in Malaysian nephrology units and primary care. It can serve as a tool for identifying communication gaps, evaluating the impact of professional training on communication skills, and tracking changes in doctor–patient interaction quality over time. Future studies could use the MyD-PCQ to evaluate communication-focused interventions or compare communication perceptions across different chronic disease populations.

Critically, the validated MyD-PCQ fills an important gap in healthcare assessment tools for Malaysia. While existing measures have been validated in other languages and cultural settings, none have previously been adapted specifically for Malay-speaking patients receiving hemodialysis. This tool can now support more systematic monitoring of communication quality, enable quality improvement initiatives, and serve as an outcome measure for interventions designed to enhance patient-centered communication in nephrology clinics and other chronic care settings.

### Limitations

This study has several limitations. First, the sample was drawn from a single center in Kelantan, which may limit the generalizability of findings to other geographic regions or healthcare settings in Malaysia. Second, illiterate patients were excluded, meaning the results may not fully capture the communication experiences of all patients receiving hemodialysis, particularly those with limited literacy or cognitive challenges. Third, the study population was predominantly Malay, potentially limiting the applicability of the findings to other ethnic groups within the multicultural Malaysian population. Additionally, as the data were based on self-reported responses, there is a possibility of social desirability and recall bias, which may affect the accuracy of the reported communication experiences.

Finally, although the MyD-PCQ demonstrated strong psychometric properties in this sample, we did not conduct measurement invariance analysis across key demographic subgroups such as gender, age, or education level. As a result, the stability and equivalence of the instrument across diverse populations could not be confirmed. Future studies should include invariance testing to establish the cross-group validity of the scale and consider validating the instrument among patients with other chronic conditions beyond hemodialysis.

## 5. Conclusions

The Malay version of the Doctor–Patient Communication Questionnaire (MyD-PCQ) demonstrated excellent construct validity and high reliability, making it a robust tool for assessing communication between doctors and patients receiving hemodialysis in Malaysia. Its availability enables healthcare providers to systematically measure and improve communication quality, contributing to more patient-centered and effective care. Future research should test the MyD-PCQ in other Malaysian settings and patient populations to further establish its generalizability and utility across diverse healthcare contexts.

## Figures and Tables

**Table 1:** Sociodemographic characteristics of respondents (n = 300).

Variables	Frequency (n)	Percent (%)
Gender		
Male	106	35.3%
Female	194	64.7%
Age group		
18–27 Years Old	50	16.7%
28–37 Years Old	38	12.7%
38–47 Years Old	40	13.3%
48–57 Years Old	70	23.3%
Over 58 Years Old	102	34.0%
Ethnicity		
Malay	142	47.3%
Chinese	72	24.0%
Indian	42	14.0%
Others	44	14.7%
Marital Status		
Bachelor	40	13.3%
Married	176	58.7%
Widow/Widower	84	28.0%
Education Level		
O level	108	36.0%
A level	80	26.7%
Diploma	48	16.0%
Degree	64	21.3%
Type of Occupation		
Government	26	8.7%
Private	60	20.0%
Self-employed	64	21.3%
Housemaker	68	22.7%
Student	16	5.3%
Not Working	66	22.0%
Income level (MYR)		
1000–4360	180	60.0%
4361–9620	90	30.0%
9621 and above	30	10.0%
Do you agree with and support the treatment provided?		
No	38	12.7%
Yes	262	87.3%
Are you satisfied with the service provided by the doctor?		
No	36	12.0%
Yes	264	88.0%
Is your doctor easily reachable?		
No	32	10.7%
Yes	268	89.3%
Is your doctor ready to treat you?		
No	28	9.3%
Yes	272	90.7%

**Table 2:** Descriptive statistics of doctor–patient communication (n = 300).

Statements	Mean (SD)	n (Percent (%))			
		1	2	3	4
Did the doctor listen to you carefully during the consultation?	2.48 (0.53)	2 (0.7)	156 (52.0)	138 (46.0)	4 (1.3)
Did the doctor allow you to talk without interrupting you?	2.50 (0.55)	3 (1.0)	148 (49.3)	144 (48.0)	5 (1.7)
Did the doctor encourage you to express yourself/talk?	2.45 (0.56)	5 (1.7)	150 (53.3)	130 (43.3)	5 (1.7)
Did the doctor examine you thoroughly?	2.52 (0.56)	4 (1.3)	142 (47.3)	147 (49.0)	7 (2.3)
Do you feel that the doctor understood you?	2.47 (0.56)	5 (1.7)	152 (50.7)	138 (46.0)	5 (1.7)
Was it easy to understand what the doctor said?	2.45 (0.57)	9 (3.0)	151 (50.3)	136 (45.3)	4 (1.3)
Do you feel you were given all the necessary information?	2.49 (0.55)	3 (1.0)	150 (53.3)	142 (47.3)	5 (1.7)
Did the doctor explain the advantages and disadvantages of the treatment or care strategy?	2.49 (0.53)	3 (1.0)	149 (49.7)	145 (48.3)	3 (1.0)
Did the doctor involve you in the decision-making?	2.49 (0.55)	6 (2.0)	143 (47.7)	148 (49.3)	3 (1.0)
In your opinion, did the doctor have a reassuring attitude and way of talking?	2.50 (0.52)	1 (0.3)	149 (49.7)	147 (49.0)	3 (1.0)
Do you think the doctor was in general respectful?	2.48 (0.56)	7 (2.3)	145 (48.3)	145 (48.3)	3 (1.0)
Did the doctor make sure that you understood his explanations and instructions?	2.48 (0.55)	6 (2.0)	146 (48.7)	145 (48.3)	3 (1.0)
Do you think the doctor told the whole truth?	2.47 (0.53)	2 (0.7)	156 (52.0)	139 (46.3)	3 (1.0)
Do you have confidence in this doctor?	2.49 (0.53)	1 (0.3)	153 (51.0)	142 (47.3)	4 (1.3)
Did the doctor reply to all your expectations and concerns?	2.50 (0.53)	4 (1.3)	142 (47.3)	152 (50.7)	2 (0.7)
Total score	37.31				

**Table 3:** Fit indices for confirmatory factor analysis of the MyD-PCQ (n = 300).

Model	$x^{2}$ (df)	$x^{2}$ /df	*p*	SRMR	RMSEA	90% CI	CFI	TLI	AIC	BIC
1	112.663 (90)	1.25	0.053	0.037	0.029	0.000, 0.045	0.982	0.979	6187.240	6298.354

CFI: comparative fit index; TLI: Tucker–Lewis fit index; AIC: Akaike information criterion; BIC: Bayesian information criterion; SRMR: standardized root mean square residual; RMSEA: root mean square error of approximation.

**Table 4:** Factor loadings for individual items and the overall reliability of the scale.

MyPDRQ Items	Factor Loading	Raykov’s Rho
Q01	0.640	0.887
Q02	0.618	
Q03	0.619	
Q04	0.608	
Q05	0.573	
Q06	0.605	
Q07	0.574	
Q08	0.571	
Q09	0.580	
Q10	0.595	
Q11	0.493	
Q12	0.584	
Q13	0.534	
Q14	0.542	
Q15	0.574	

## Data Availability

The data presented in this study are available upon request from the corresponding author. The data are not publicly available due to privacy or ethical restrictions.

## Supplementary Materials

The following supporting information can be downloaded at https://www.mdpi.com/article/10.3390/healthcare13162037/s1, Table S1: Inter-Item Correlation Matrix for the MyD-PCQ (n = 300). Table S2: Skewness and Kurtosis Values for Each Item in the MyD-PCQ.

## Abbreviations

The following abbreviations are used in this manuscript:

MyD-PCQ	Malay version of the Doctor–Patient Communication Questionnaire
CFA	Confirmatory factor analysis
CFI	Comparative fit index
TLI	Tucker–Lewis index
AIC	Akaike information criterion
BIC	Bayesian information criterion
SRMR	Standardized root mean square residual
RMSEA	Root mean square error of approximation

## References

[1] Selinger C.P. (2009). The right to consent: Is it absolute?. Br. J. Med. Pract..

[2] Browning P.E., Longe J.L. (2018). Professional patient relationship. How to Reference Books.

[3] Curran J. (2007). The doctor, his patient, and the illness. BMJ.

[4] Goold S.D., Lipkin M. (1999). The doctor–patient relationship. J. Gen. Intern. Med..

[5] Kelley J.M., Kraft-Todd G., Schapira L., Kossowsky J., Riess H. (2014). The influence of the patient–clinician relationship on healthcare outcomes: A systematic review and meta-analysis of randomized controlled trials. PLoS ONE.

[6] Dwolatzky T., Clarfield A.M., Schulz R. (2006). Doctor–patient relationships. Encyclopedia of Aging.

[7] Duffy F.D., Gordon G.H., Whelan G., Cole-Kelly K., Frankel R. (2004). Assessing competence in communication and interpersonal skills: The Kalamazoo II Report. Acad. Med..

[8] Arora N.K. (2003). Interacting with cancer patients: The significance of physicians’ communication behavior. Soc. Sci. Med..

[9] Tongue J.R., Epps H.R., Forese L.L. (2005). Communication skills for patient-centered care: Research-based, easily learned techniques for medical interviews that benefit orthopedic surgeons and their patients. J. Bone Jt. Surg. Am..

[10] Lee S.J., Back A.L., Block S.D., Stewart S.K. (2002). Enhancing physician–patient communication. Hematol. Am. Soc. Hematol. Educ. Program..

[11] Kindler C.H., Szirt L., Sommer D., Hausler R., Langewitz W. (2005). A quantitative analysis of anesthetist–patient communication during the pre-operative visit. Anaesthesia.

[12] Middleton S., Gattellari M., Harris J.P., Ward J.E. (2006). Assessing surgeons’ disclosure of risk information before carotid endarterectomy. ANZ J. Surg..

[13] Foy R., Walker A., Ramsay C., Penney G., Grimshaw J., Francis J. (2005). Frequency of patient–physician contact in chronic kidney disease care and achievement of clinical performance targets. Int. J. Qual. Health Care.

[14] Pinheiro J., Maia M., Alves H. (2013). The physician–patient relationship in dialysis. Port. J. Nephrol. Hypert.

[15] Argentero P., Dell’Olivo B., Ferretti M.S. (2008). Staff burnout and satisfaction with the quality of dialysis care. Am. J. Kidney Dis..

[16] Kovac J.A., Patel S.S., Peterson R.A., Kimmel P.L. (2002). Patient satisfaction with care and behavioral compliance in end-stage renal disease patients treated with hemodialysis. Am. J. Kidney Dis..

[17] Jha A.K., Orav E.J., Zheng J., Epstein A.M. (2008). Patients’ perception of hospital care in the United States. N. Engl. J. Med..

[18] Mazairac A.H., de Wit G.A., Penne E.L., van der Weerd N.C., de Jong B., Grooteman M.P., van den Dorpel M.A., Buskens E., Dekker F.W., Nubé M.J. (2011). Changes in quality of life over time: Dutch hemodialysis patients and general population compared. Nephrol. Dial. Transplant..

[19] Manzoor F., Wei L., Hussain A., Asif M., Shah S.I., Akram M.N. (2019). Patient satisfaction with health care services: An application of physician’s behavior as a moderator. Int. J. Environ. Res. Public Health.

[20] Ridd M., Shaw A., Lewis G., Salisbury C. (2009). The patient–doctor relationship: A synthesis of the qualitative literature on patients’ perspectives. Br. J. Gen. Pract..

[21] Huggard P. (2003). Compassion fatigue: How much can I give?. Med. Educ..

[22] Smith J.A. (2011). Evaluating the contribution of interpretive phenomenological analysis. Health Psychol. Rev..

[23] Bennett J., O’Donovan D. (2001). Substance misuse by doctors, nurses, and other healthcare workers. Curr. Opin. Psychiatry.

[24] Tyssen R., Vaglum P. (2002). Mental health problems among young doctors: An updated review of prospective studies. Harv. Rev. Psychiatry.

[25] Ball L., Maben J., Griffiths P. (2018). Questionnaires that measure the quality of relationships between patients and primary care providers: A systematic review. BMC Health Serv. Res..

[26] Carretta E., Bond T.G., Cappiello G., Fantini M.P. (2017). Looking through the patients’ eyes: Measuring patient satisfaction in a public hospital. J. Patient Exp..

[27] Xie Z., Or C. (2017). Associations between waiting times, service times, and patient satisfaction in an endocrinology outpatient department: A time study and questionnaire survey. Health Serv. Res..

[28] Silverman J., Kurtz S., Draper J. (2006). Skills for Communicating with Patients. Ann. R. Coll. Surg. Engl..

[29] Sustersic M., Jeannin A., Sandoz V., Cerutti B., Junod Perron N. (2018). A scale assessing doctor–patient communication in a context of acute conditions based on a systematic review. PLoS ONE.

[30] Freburger J.K., Callahan L.F., Currey S.S., Anderson L.A. (2003). Use of the Trust in Physician Scale in patients with rheumatic disease: Psychometric properties and correlates of trust in the rheumatologist. Arthritis Rheum..

[31] Hatcher R.L., Gillaspy J.A. (2006). Development and validation of a revised short version of the Working Alliance Inventory. Psychother. Res..

[32] Munder T., Wilmers F., Leonhart R., Linster H.W., Barth J. (2010). Working Alliance Inventory-Short Revised (WAI-SR): Psychometric properties in outpatients and inpatients. Clin. Psychol. Psychother..

[33] Andersson L.A., Dedrick R.F. (1990). Development of the Trust in Physician Scale: A measure to assess interpersonal trust in patient–physician relationships. Psychol. Rep..

[34] Thom D.H., Ribisl K.M., Stewart A.L., Luke D.A. (1999). Further validation and reliability testing of the Trust in Physician Scale. Med. Care.

[35] Ministry of Health Malaysia (2017). Malaysia Health System Performance. http://www.moh.gov.my/.

[36] Wild D., Grove A., Martin M., Eremenco S., McElroy S., Verjee-Lorenz A., Erikson P., ISPOR Task Force for Translation and Cultural Adaptation (2005). Principles of good practice for the translation and cultural adaptation process for patient-reported outcomes (PRO) measures: Report of the ISPOR task force for translation and cultural adaptation. Value Health.

[37] Martinez S.M., Ainsworth B.E., Elder J.P. (2008). A review of physical activity measures used among US Latinos: Guidelines for developing culturally appropriate measures. Ann. Behav. Med..

[38] Tabachnick B.G., Fidell L.S. (2013). Using Multivariate Statistics.

[39] Cook D.A., Beckman T.J. (2006). Current concepts in validity and reliability for psychometric instruments: Theory and application. Am. J. Med..

[40] Raykov T. (2001). Estimation of congeneric scale reliability using covariance structure analysis with nonlinear constraints. Br. J. Math. Stat. Psychol..

[41] Pituch K.A., Stevens J.P. (2015). Applied Multivariate Statistics for the Social Sciences: Analyses with SAS and IBM’s SPSS.

[42] Schreiber J.B., Nora A., Stage F.K., Barlow E.A., King J. (2006). Reporting structural equation modeling and confirmatory factor analysis results: A review. J. Educ. Res..

[43] Brown T.A. (2014). Confirmatory Factor Analysis for Applied Research.

[44] Blais M.A. (2004). Development of an inpatient treatment alliance scale. J. Nerv. Ment. Dis..

