# Supplementary material for: Translation and Validation of the Malay Doctor–Patient Communication Questionnaire: A Cross-Sectional Study Among Patients Receiving Hemodialysis in Kelantan, Malaysia
Source: Healthcare (Basel). 2025 Aug 18;13(16):2037. doi: 10.3390/healthcare13162037 (PMC12386149; doi:10.3390/healthcare13162037)
Supplement: Supplementary file 1 [file healthcare-13-02037-s001.zip › healthcare-3770177-supplementary.pdf]

Table S1: Inter-Item Correlation Matrix for the MyD-PCQ (n = 300)

	**Q01**	**Q02**	**Q03**	**Q04**	**Q05**	**Q06**	**Q07**	**Q08**	**Q09**	**Q10**	**Q11**	**Q12**	**Q13**	**Q14**	**Q15**
**Q01**	1	0.456	0.444	0.367	0.345	0.356	0.377	0.356	0.422	0.366	0.274	0.372	0.377	0.343	0.357
**Q02**	0.456	1	0.389	0.351	0.399	0.336	0.385	0.377	0.353	0.408	0.339	0.392	0.342	0.284	0.253
**Q03**	0.444	0.389	1	0.380	0.292	0.394	0.356	0.314	0.335	0.403	0.351	0.286	0.410	0.335	0.371
**Q04**	0.367	0.351	0.380	1	0.304	0.349	0.330	0.376	0.364	0.395	0.267	0.412	0.399	0.353	0.332
**Q05**	0.345	0.399	0.292	0.304	1	0.314	0.312	0.335	0.378	0.344	0.394	0.393	0.344	0.278	0.325
**Q06**	0.356	0.336	0.394	0.349	0.314	1	0.345	0.336	0.336	0.379	0.269	0.350	0.430	0.357	0.413
**Q07**	0.377	0.385	0.356	0.330	0.312	0.345	1	0.264	0.353	0.294	0.274	0.380	0.375	0.296	0.377
**Q08**	0.356	0.377	0.314	0.376	0.335	0.336	0.264	1	0.311	0.412	0.231	0.261	0.448	0.331	0.322
**Q09**	0.422	0.353	0.335	0.364	0.378	0.336	0.353	0.311	1	0.251	0.276	0.328	0.354	0.332	0.378
**Q10**	0.366	0.408	0.403	0.395	0.344	0.379	0.294	0.412	0.251	1	0.316	0.314	0.424	0.304	0.259
**Q11**	0.274	0.339	0.351	0.267	0.394	0.269	0.274	0.231	0.276	0.316	1	0.345	0.260	0.217	0.298
**Q12**	0.372	0.392	0.286	0.412	0.393	0.350	0.380	0.261	0.328	0.314	0.345	1	0.325	0.326	0.351
**Q13**	0.377	0.342	0.410	0.399	0.344	0.430	0.375	0.448	0.354	0.424	0.260	0.325	1	0.341	0.344
**Q14**	0.343	0.284	0.335	0.353	0.278	0.357	0.296	0.331	0.332	0.304	0.217	0.326	0.341	1	0.378
**Q15**	0.357	0.253	0.371	0.332	0.325	0.413	0.377	0.322	0.378	0.259	0.298	0.351	0.344	0.378	1

Table S2: Skewness and Kurtosis Values for Each Item in the MyD-PCQ

Item	Skewness (SE)	Kurtosis (SE)
Q01	0.209 (0.141)	-1.037 (0.281)
Q02	0.107 (0.141)	-0.869 (0.281)
Q03	0.198 (0.141)	-0.646 (0.281)
Q04	0.072 (0.141)	-0.653 (0.281)
Q05	0.092 (0.141)	-0.693 (0.281)
Q06	-0.064 (0.141)	-0.556 (0.281)
Q07	0.134 (0.141)	-0.863 (0.281)
Q08	0.027 (0.141)	-1.065 (0.281)
Q09	-0.148 (0.141)	-0.789 (0.281)
Q10	0.111 (0.141)	-1.321 (0.281)
Q11	-0.147 (0.141)	-0.729(0.281)
Q12	-0.109 (0.141)	-0.796(0.281)
Q13	0.160 (0.141)	-1.159 (0.281)
Q14	0.213 (0.141)	-1.178 (0.281)
Q15	-0.155 (0.141)	-1.058 (0.281)
